# Epithelial and Neural Cadherin in Mammalian Fertilization: Studies in the Mouse Model

**DOI:** 10.3390/cells11010102

**Published:** 2021-12-29

**Authors:** Gustavo Luis Verón, María Florencia Veiga, Mónica Cameo, Clara Isabel Marín-Briggiler, Mónica Hebe Vazquez-Levin

**Affiliations:** 1 Laboratorio de Estudios de Interacción Celular en Reproducción y Cáncer, Instituto de Biología y Medicina Experimental (IBYME), Consejo Nacional de Investigaciones Científicas y Técnicas (CONICET), Fundación IBYME (FIBYME), Buenos Aires C1428ADN, Argentina; gustavo.veron@live.com (G.L.V.); mariaflorencia.veiga@gmail.com (M.F.V.); clarisamarin@gmail.com (C.I.M.-B.); 2 BioAplicada—Laboratorio de Cultivos Celulares y Estudios Especiales, Buenos Aires C1430EYV, Argentina; mcameo@bioaplicada.com

**Keywords:** epithelial cadherin, neural cadherin, in vitro fertilization, spermatozoa, oocyte

## Abstract

Successful mammalian fertilization requires a well-orchestrated sequence of molecular events leading to gamete fusion. Since this interaction involves Ca^2+^-dependent adhesion events, the participation of the Ca^+2^-dependent cell-cell adhesion proteins Epithelial (E-cad) and Neural (N-cad) cadherin is envisaged. We have previously reported the expression of E-cad and N-cad in human gametes and showed evidence of their involvement in sperm-oocyte adhesion events leading to fertilization. To overcome ethical limitations associated with the use of human gametes in fertilization-related studies, the mouse has been selected worldwide as the experimental model for over 4 decades. Herein, we report a detailed study aimed at characterizing the expression of E-cad and N-cad in murine gametes and their involvement in murine fertilization using specific antibodies and blocking peptides towards both adhesion proteins. E-cad and N-cad protein forms, as well as other members of the adhesion complex, specifically β-catenin and actin, were identified in spermatozoa, cumulus cells and oocytes protein extracts by means of Western immunoblotting. In addition, subcellular localization of these proteins was determined in whole cells using optical fluorescent microscopy. Gamete pre-incubation with anti-E-cad (ECCD-1) or N-cad (H-63) antibodies resulted in decreased (*p* < 0.05) In Vitro Fertilization (IVF) rates, when using both cumulus-oocytes complexes and cumulus-free oocytes. Moreover, IVF assays done with denuded oocytes and either antibodies or blocking peptides against E-cad and N-cad led to lower (*p* < 0.05) fertilization rates. When assessing each step, penetration of the cumulus mass was lower (*p* < 0.05) when spermatozoa were pre-incubated with ECCD-1 or blocking peptides towards E-cad or towards both E- and N-cad. Moreover, sperm-oolemma binding was impaired (*p* < 0.0005) after sperm pre-incubation with E-cad antibody or blocking peptide towards E-cad, N-cad or both proteins. Finally, sperm-oocyte fusion was lower (*p* < 0.05) after sperm pre-incubation with either antibody or blocking peptide against E-cad or N-cad. Our studies demonstrate the expression of members of the adherent complex in the murine model, and the use of antibodies and specific peptides revealed E-cad and N-cad participation in mammalian fertilization.

## 1. Introduction

Fertilization is an exceptional multistep process that involves two highly differentiated cells: the spermatozoon and the oocyte. After spermatogenesis, spermatozoa go through structural and functional modifications in the epididymis in a process known as sperm maturation [1,2,3,4]. At ejaculation, mature spermatozoa are placed into the female reproductive tract, where they undergo profound changes required to fully develop their fertilizing capacity in a process denoted as capacitation [5,6]. Once they arrive at the oocyte vicinity, spermatozoa penetrate the cumulus oophorus and undergo the acrosomal exocytosis (AE), a process in which the plasma membrane and outer acrosomal membrane fuse, releasing the acrosomal content and exposing the fusogenic region. Afterwards, spermatozoa penetrate the *zona pellucida* (ZP) and finally bind and fuse with the oocyte plasma membrane (oolemma) [7,8,9,10,11].

In the last 40 years, great efforts have been made to identify gamete proteins involved in fertilization and several components have been reported using cellular, biochemical, immunological, and molecular approaches [12,13,14,15]. However, the molecular bases of this complex process have not been completely elucidated. Cadherins belong to a Ca^2+^-dependent adhesion cell membrane glycoprotein superfamily [16], involved in homotypic (same cell) and homophilic (same cadherin) cell-cell adhesion events, being Epithelial cadherin (E-cad; uvomorulin; CDH1; L-CAM, ARC-1) the founder member [17,18]. E-cad is a 120 kDa glycoprotein composed of an extracellular, a single transmembrane and a cytoplasmic domain. While the extracellular domain participates in cell-cell adhesion, the cytoplasmic domain is involved in intracellular cell signaling and links E-cad to filamentous actin (F-actin) through adaptor molecules, among them β-catenin [18]. Another member of the classical cadherin family is Neural cadherin (N-cad, CDH2), a 135 kDa transmembrane protein first identified as a neural cells adhesion molecule, although later was found to be expressed in several tissues [19]. Participation of E-cad and N-cad in cell-cell adhesion and signal transduction events has been extensively investigated in embryonic and somatic cells in health and disease [20,21,22,23,24,25,26]. While its structure resembles that of E-cad, N-cad mediates homotypic binding, although during tumor progression it also participates in heterotypic adhesion events involving E-cad on the cancer cell membrane and N-cad on the fibroblast membrane [27].

**Figure 1:**
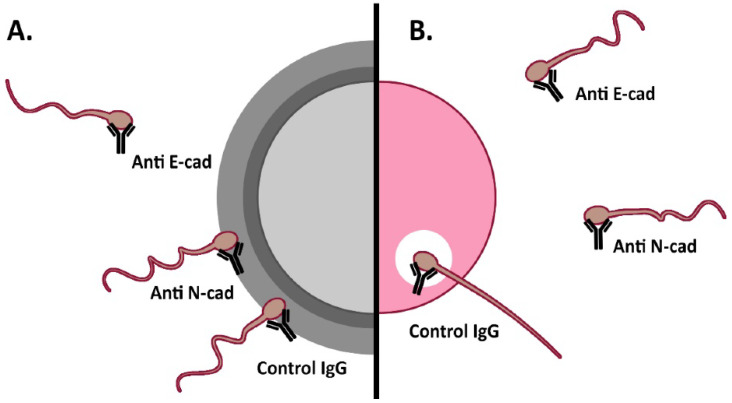
Schematic representation of evidence gathered on the participation of E-cad and N-cad in human sperm-oocyte interaction. (**A**). Sperm–ZP interaction was significantly inhibited after sperm pre-incubation with anti E-cad antibodies (Hemizona assay). (**B**). Sperm interaction with the oolemma: Sperm penetration of ZP-free hamster oocytes was significantly impaired after human sperm pre-incubation with anti E-cad or N-cad antibodies (SPA assay).

Contrasting with the information available about E-cad and N-cad expression and function(s) in somatic and embryonic cells, their expression in germ cells and their role in mammalian fertilization remains scarce. Since mammalian fertilization involves Ca^2+^-dependent adhesion events [28], participation of these cell-cell adhesion proteins is envisaged. Our group has previously reported expression of E-cad and N-cad in human oocytes and spermatozoa, and has shown evidence of E-cad and N-cad participation in sperm-oocyte interaction events [29,30,31]. Specifically, spermatozoa incubated with anti-E-cad antibodies showed impaired binding to homologous ZP by means of the hemizona assay (HZA; Figure 1). In addition, presence of these antibodies inhibited the penetration of human spermatozoa to ZP-free hamster oocytes [29]. In contrast, sperm incubation with anti N-cad antibodies did not affect their ability to interact with homologous ZP in the HZA; but presence of anti-N-cad antibodies led to a significant reduction in the percentage of penetrated ZP-free hamster oocytes [30]. Despite both proteins being involved in homophilic interactions, previous studies did not assess human E-cad or N-cad role in homologous fertilization due to ethical restrictions.

The mouse model has been extensively used for over 40 years to develop studies aimed to understand the molecular basis of fertilization. Studies on E-cad and N-cad expression during murine gonad development and in adult testis, ovary and epididymis and has been recently thoroughly reviewed [12]. Moreover, we have previously reported evidence of their expression in murine gametes [31].

The present investigation was done to study E- and N-cad expression in spermatozoa and cells from the Cumulus-Oocyte Complex (COC) using the murine model. In addition, the studies aimed at evaluating the involvement of both adhesion proteins in homologous fertilization by performing in vitro gamete interaction assays in the presence of blocking antibodies and peptides towards each and both adhesion proteins.

## 2. Materials and Methods

### 2.1. Reagents

Unless otherwise indicated, analytical and tissue culture grade reagents were purchased from Sigma Chemical Co. (St. Louis, MO, USA), and electrophoresis reagents were products of BioRad (Richmond, CA, USA).

Mouse E-cad was immunodetected using a polyclonal antibody H-108 (rabbit; against residues 600–707 of human E-cad extracellular cadherin domain 5; Santa Cruz Biotech., Santa Cruz, CA, USA), and monoclonal antibodies 610181 (mouse; against human E-cadherin residues 773–791 of cytoplasmic domain; BD Biosciences, San Diego, CA, USA), and ECCD-1 (rat; against mouse E-cad extracellular cadherin domain 1; Zymed-Invitrogen, South San Francisco, CA, USA). To immunodetect N-cad, the polyclonal antibody H-63 (rabbit, raised against amino acids 450–512 of the human N-cad extracellular domain; Santa Cruz Biotech., Santa Cruz, CA, USA) was applied for Western immunoblotting, immunocytochemistry and gamete interaction assays. Antibodies towards β-catenin (610153, BD Biosciences; 06-734, Upstate, Lake Placid, NY, USA) and actin (I-19, Sigma) were used in Western immunoblotting protocols of mouse spermatozoa, cumulus cells and oocytes. In addition, fluorescence localization of β-catenin and F-actin was done in whole mouse spermatozoa, cumulus cells and oocytes using the same antibody towards the adaptor protein and FITC-Phalloidin to localize F-actin.

Cy3-labeled anti-mouse or anti-rabbit IgG secondary antibody (Chemicon-Millipore, Billerica, MA, USA) was used for immunocytochemistry protocols, as specifically required. Horse-radish peroxidase (HRP)-conjugated goat anti-rabbit or anti-mouse IgGs were used in Western immunoblotting assays (Thermo Fisher Sci., Carslbad, CA, USA). Purified IgG from normal rat, rabbit and mouse sera were obtained from Sigma and used in control experiments. Staining of DNA content with Hoechst 33342 (bis-benzimide) was performed when indicated. Vectashield antifade solution was purchased from Vector Labs.

Peptides used for gamete interaction assays were as follows; for E-cad: AKYILYSHAVSSNGEAV and VLYSYHASNIVEKSAGA (blocking and scramble, respectively; [32], for N-cad: H-SWTLYTPSGQSK-NH2 and H-SRTLYTPSGQSK-NH2 (blocking and control, respectively; [33], and for both E-cad and N-cad: H-SWELYYPLRANL-NH2 and H-SRELYYPLRANL-NH2 (blocking and control, respectively; [34]). Each blocking peptide recognizes the adhesion protein extracellular domain. All peptides were produced upon request by GenScript (Piscataway, NJ, USA).

### 2.2. Gamete Handling

Animals were treated in compliance with the National Institute of Health Guide for the Care and Use of Laboratory Animals [35], and this study was approved by the CICUAL (Institutional Committee for the Laboratory Animal Use and Care; protocol 012/2017 approved on 25 April 2017) of the Instituto de Biología y Medicina Experimental (IBYME). *Cauda* epididymal spermatozoa, as well as COCs, were retrieved from mice for immunocytochemistry, In Vitro Fertilization (IVF) and sperm-oolemma interaction assays. Motile epididymal spermatozoa were obtained by swim-out from *cauda* epididymides of adult CF1 mice. Briefly, *cauda* epididymides were placed in IVF culture medium droplets for 10 min to enable sperm release; sperm motility was evaluated subjectively. COCs were collected from CF1 female mice (at least 8 weeks old) subjected to standard ovarian stimulation [36]. For assays performed with denuded oocytes, COCs were incubated with 3 mg/mL hyaluronidase for 2–3 min, and Metaphase II oocytes (identified by the presence of the first polar body; MII) were washed to remove hyaluronidase and cumulus cells excess. To remove the ZP, oocytes were placed in Tyrode’s acid solution for 5 s, followed by a wash with IVF culture medium [37].

### 2.3. Protein Extraction and Western Immunoblotting

*Cauda* epididymal spermatozoa were placed at 37 °C for 10 min in 200 µL droplets of IVF culture medium supplemented with 4 mg/mL of polyvynilpirrolidone and 4 mM EGTA under oil. Spermatozoa were recovered and centrifuged (300× *g* for 10 min). The pellet was resuspended in lysis buffer [38], incubated for 2 h in ice and centrifuged (10 min, 12,000× *g*) again. The supernatant containing the extracted proteins was supplemented with Laemmli sample buffer and stored at −20 °C until analysis. Protein homogenates were also prepared from adult testis and epididymis using standard procedures and supplemented with Laemmli sample buffer and stored as indicated above. Cumulus cells and Metaphase-II oocytes were also resuspended in Laemmli sample buffer and stored at −20 °C. Protein concentration was determined by means of the Bradford assay (BioRad). Protein samples were boiled for 5–10 min, supplemented with 5% beta-mercaptoethanol and subjected to SDS-PAGE in 10% polyacrylamide gels with 0.1% SDS under reducing conditions [39]. Protein molecular weight (MW) was estimated by comparison with protein standards (Molecular Weight Markers, MWM; Broad Range; BioRad). Proteins were transferred to nitrocellulose membranes (Hybond-ECL, Amersham/GE, Buckinghamshire, Great Britain) [40]. Membranes were placed in blocking solution (5% skimmed milk in PBS containing 0.02% Tween-20) for 1 h, followed by overnight incubation at 4 °C with primary antibodies against: E-cad (610181: 0.25 µg/mL), N-cad (H63: 2 µg/mL), β-catenin (610153: 0.5 µg/mL); or actin (I-19: 0.06 µg/mL). Control experiments were run using mouse or rabbit IgG added in replacement and at the same concentration of the primary antibody. Membranes were washed, placed for 1 h at room temperature with anti-mouse or anti-rabbit horseradish peroxidase-conjugated IgGs (1:1000 in blocking solution), and developed with enhanced chemiluminiscence (ECL kit, Amersham/GE).

### 2.4. Sperm Capacitation and Acrosomal Exocytosis

Swim-out was performed for 10 min at 37 °C in a IVF medium supplemented with 3 mg/mL BSA. Spermatozoa were recovered and capacitated at a 1 × 10^6^ spermatozoa/mL concentration in IVF culture medium for 90 min at 37 °C and 5% CO_2_ in air atmosphere. Spermatozoa were exposed to 10 µM Ca^2+^-ionophore A23187 in the last 15 min to induce AE.

To assess the acrosomal status, spermatozoa were incubated with the lectin *Pisum sativum* agglutinin labelled with FITC (PSA-FITC). Briefly, spermatozoa were permeabilized with 100% methanol for 20 s, incubated for 1 h at room temperature with 50 µg/mL of PSA-FITC, washed with Dulbecco-PBS (D-PBS), mounted, and analysed. At least 200 cells were evaluated in each experimental condition. Acrosome-intact spermatozoa were scored when presenting bright staining over the acrosomal cap, and acrosome-reacted spermatozoa were scored when presenting a signal over the equatorial segment or absence of fluorescent label [41]. The sperm acrosomal status was assessed with a confocal laser scanning microscope (Olympus FV1000; Tokyo, Japan) using 600× magnification; images were analyzed using the Image J software. At least 200 spermatozoa were counted in each experimental condition.

### 2.5. Immunocytochemistry

Spermatozoa were fixed in 2% formaldehyde in D-PBS for 4 min, washed with D-PBS, concentrated by cell centrifugation (3000 rpm, 3 min), loaded onto microscope glass slides pre-treated with polylysine, and allowed to dry. Cells were washed in D-PBS for 5 min and placed for 30 min in PBS supplemented with 40 mg/mL BSA to block non-specific binding sites. Spermatozoa were incubated overnight at 4 °C with specific antibodies (E-cad H-108 4 μg/mL; N-cad H-63 4 μg/mL) in PBS supplemented with 20 mg/mL BSA. After washing, cells were incubated with Cy3-labeled anti-rabbit secondary antibody in D-PBS (1:500) for 1 h at room temperature. Slides were washed and mounted with Vectashield. In some cases, this procedure was combined with PSA-FITC staining to assess acrosomal status.

Cumulus cells and MII oocytes with or without ZP were fixed in 2% formaldehyde supplemented with 0.6 µM Placlitaxel in PBS for 2 h at room temperature. Cells were then placed for 45 min in blocking buffer (0.5% BSA, 1% goat serum, 100 mM glycine, 0.01% Triton X-100 in PBS) followed by an overnight incubation at 4 °C with anti E-cad (H-108; 4 μg/mL) or N-cad (H-63; 4 μg/mL) antibodies in IVF media containing 20 mg/mL BSA. Cells were washed and incubated with Cy3-labeled anti-rabbit secondary antibody in D-PBS (1:500), washed, and mounted with Vectashield. In all cases, control procedures were carried out in parallel using the same concentration of purified IgG (negative control), followed by DNA staining with Hoechst 33342. Stained cells were observed with confocal laser scanning microscope (Olympus FV1000; Tokyo, Japan).

### 2.6. Sperm-Oocyte Interaction Assays

CF1 female mice were super-ovulated and COCs were harvested for gamete interaction procedures. At the end of the assays, a group of oocytes was stained with 0.25% Trypan Blue or propidium iodide in PBS to assess their viability.

#### 2.6.1. IVF

Protocols were as previously reported by our group with some modifications [36]. Briefly, *cauda* epididymal spermatozoa were capacitated in IVF medium with 3 mg/mL BSA at 37 °C in an atmosphere of 5% CO_2_ in air for 90 min. In the last 60 min of incubation, anti E-cad or N-cad antibodies (20 µg/mL ECCD-1 or H-63, respectively), or control IgG (20 µg/mL rat or rabbit IgG, respectively) were added to the sperm suspension. This was also repeated with E-cad and/or N-cad blocking peptides (500 µM). COCs or MII oocytes were added to a 50 µL droplet of IVF medium containing 1 × 10^5^ pre-treated spermatozoa. Gamete co-incubation was carried out for 3 h at 37 °C and 5% CO_2_ in air. Fertilization was confirmed by the presence of both male and female pronucleae (2PN, 2PB). Results were expressed as percentages of fertilized oocytes and calculated as: (Number of fertilized oocytes/Number of inseminated oocytes) × 100.

#### 2.6.2. Cumulus Penetration Assay

Spermatozoa were incubated with anti E-cad or N-cad antibodies or blocking peptides (20 µg/mL, 500 µM, respectively) or control reagents (IgGs 20 µg/mL, control peptides 500 µM) during the last 60 min of capacitation. Afterwards, spermatozoa nuclei were stained using Hoechst 33342 (20 μg/mL) for 10 min and then washed. COCs were added to an IVF medium droplet containing the treated spermatozoa and co-incubated for 15 min. Non-specifically bound spermatozoa were removed by extensive washing, and COCs were fixed in 2% formaldehyde supplemented with 0.6 µM Placlitaxel in PBS for 2 h at room temperature, washed with PBS 3 times (2 min each) at room temperature and mounted in a clean slide with PBS and Vectashield to assess the number of spermatozoa penetrating the cumulus layer [42].

#### 2.6.3. Sperm-Oolemma Binding and Fusion Assays

ZP-free oocytes were added to an IVF medium droplet containing spermatozoa previously incubated with anti E- or N-cad antibodies or blocking peptides (20 µg/mL, 500 µM, respectively) or control reagents (IgGs 20 µg/mL, peptides 500 µM) and co-incubated for 1 h. Non-specifically bound spermatozoa were removed by extensive washing. Oocytes were fixed, stained (Hoechst 33342), mounted and analysed to assess the number of spermatozoa bound to the oolemma (binding assay) or the percentage of oocytes with decondensed sperm heads (fusion assay) [43].

### 2.7. Statistical Analysis

Experiments were run at least three times in all cases. Data were expressed as Mean ± Standard Deviation of the Mean (SDM), unless otherwise stated. All statistical data analysis was done using GraphPad Prism 5 (GraphPad Software, San Diego, CA, USA). Differences in the number of spermatozoa bound to the oolemma were determined using the Mann–Whitney test. IVF and sperm-oocyte fusion rates were evaluated using the Chi-squared test. Differences were considered significant when *p* < 0.05.

## 3. Results

### 3.1. Immunodetection of E-cad and N-cad and Other Members of the Adherent Complex in Testis, Epididymis, and Cauda Epididymal Spermatozoa

**Figure 2:**
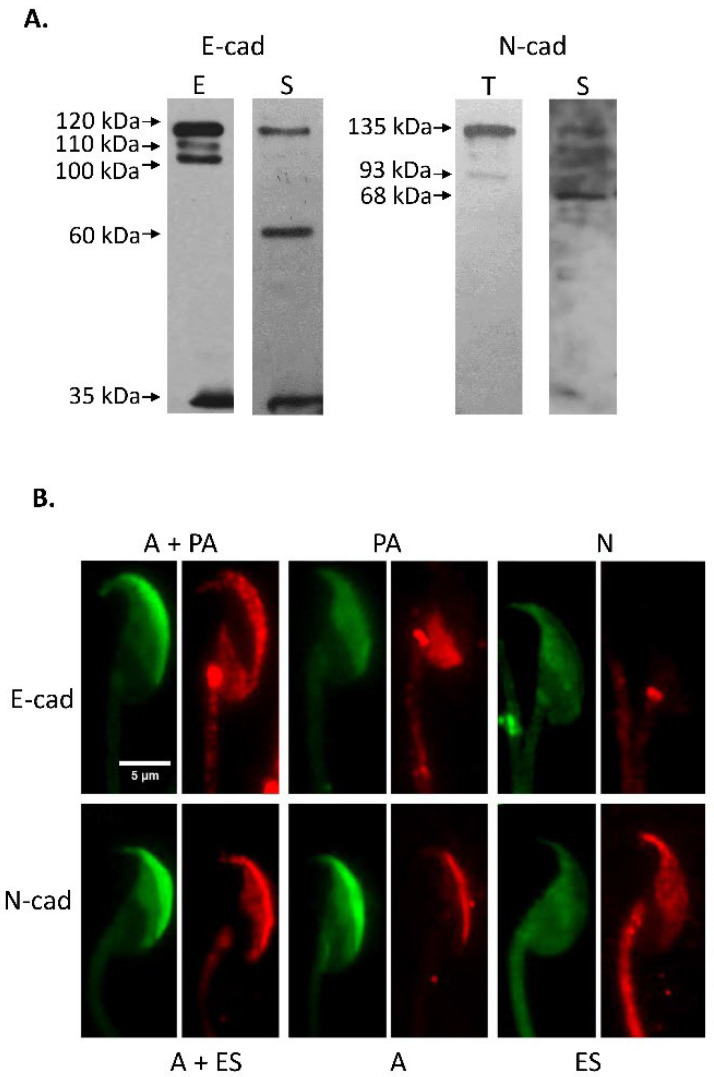
Immunodetection of E-cad and N-cad in mouse testis, epididymis and spermatozoa. (**A**). SDS-PAGE followed by Western immunoblotting of murine testis (T), epididymis (E) and spermatozoa (S) protein extracts, was done using anti E-cad and antiN-cad antibodies. E-cad protein forms of 120, 110, 100, 60 and 35 kDa were found, whereas N-cad protein forms of 135, 93 and 68 kDa were immunodetected. The signal was specific since it was absent when specific primary antibodies were replaced by control IgG (data not shown). (**B**). Fluorescence microscopy of fixed *cauda* epididymal spermatozoa. Immunodetection of E-cad, N-cad using specific antibodies. Spermatozoa depicted a specific signal for E-cad in the sperm acrosomal and post-acrosomal region (A + PA), only in the post-acrosomal region (PA), or show absence of signal (N). N-cad was immunolocalized at the acrosomal region and equatorial segment (A + ES), and only at the acrosomal region (A) or equatorial segment (ES). Green: PSA-FITC; red: Cy3-labelled secondary antibody. Bar: 5 µm.

**Figure 3:**
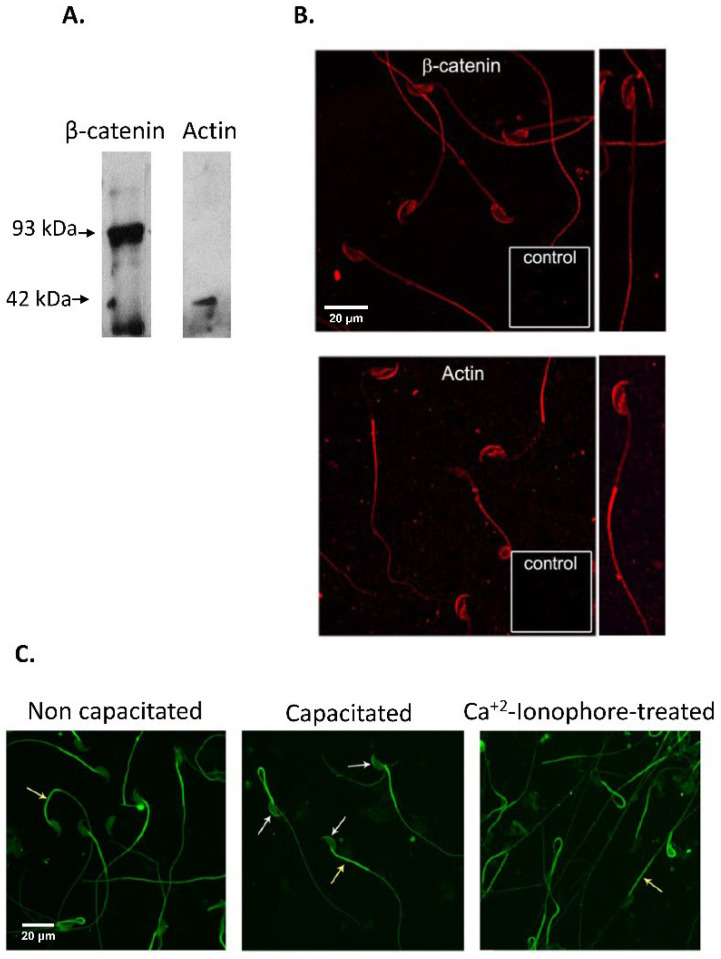
Immunodetection of β-catenin and actin in mouse spermatozoa. (**A**). SDS-PAGE followed by Western immunoblotting of murine sperm protein extracts was done using anti β-catenin and actin antibodies. A β-catenin protein form of 93 kDa, and a 42 kDa actin form were immunodetected. The signal was specific since it was absent when specific primary antibodies were replaced by control IgG (data not shown). (**B**). Fluorescence microscopy of fixed *cauda* epididymal spermatozoa and immunodetection of β-catenin and actin using specific antibodies. Spermatozoa depicted a specific signal for β-catenin and actin in the sperm acrosomal and post-acrosomal region and in the equatorial segment (Red: Cy3-labelled secondary antibody). (**C**). Fluorescence microscopy of fixed *cauda* non-capacitated, capacitated and Ca^+2^-ionophore-treated epididymal spermatozoa and immunodetection of F-actin using FITC-Phalloidin. Spermatozoa depicted a specific signal for F-actin in the acrosomal cap and flagellum (white arrows) (Green: FITC-Phalloidin). Bar: 20 µm.

Initial studies were carried out with spermatozoa recovered from the *cauda* epididymis of CF1 adult mice. Sperm count, progressively motile and acrosome-reacted spermatozoa were found within acceptable values in all samples studied (sperm count: 14.0 ± 2.3 million spermatozoa; progressive motility: 77.7 ± 1.7%; acrosome-reacted spermatozoa: 11.0 ± 1.5%). Western immunoblotting analysis was done with specific antibodies against E- and N-cad on sperm protein extracts and whole epididymal and testicular tissue homogenates. These studies led to the identification of specific protein forms of the expected molecular weight, 120 kDa for E-cad and 135 kDa for N-cad, in the tissue and gamete extracts, accompanied with protein band of lower MW (E-cad: 110, 100, 60 and 35 kDa; N-cad: 93 and 68 kDa) (Figure 2A). Moreover, immunodetection of β-catenin and actin in sperm extracts identified protein forms of 93 kDa for β-catenin, and 42 kDa for actin (Figure 3A).

Immunolocalization studies of E-cad and N-cad were done by fluorescent immunocytochemistry with specific antibodies towards E-cad and N-cad, followed by PSA-FITC staining. Representative images of protein localization of intact and acrosome-reacted spermatozoa are shown in Figure 2B. These studies revealed a strong signal for E-cad in the acrosomal and post-acrosomal regions of acrosome intact spermatozoa (A + PA), whereas N-cad displayed a signal over the acrosomal region or both the acrosomal cap and equatorial segment (A, and A + ES). On the other hand, cells classified as acrosome-reacted lost the E-cad and N-cad signal in the acrosomal cap (pattern A) but remained immunoreactive to E-cad in the post-acrosomal region (PA) and to N-cad in the equatorial segment (ES).

Immunolocalization studies of β-catenin and actin revealed a signal in the acrosomal cap, equatorial segment and post-acrosomal region, in addition to a signal in the flagellar principal piece (Figure 3B). Taking into account that actin polymerization is a key factor to define the strength of the cadherin-mediated interaction, F-actin localization was evaluated in non-capacitated, capacitated and ionophore-treated spermatozoa, finding a strong signal in the sperm flagella in all conditions evaluated, while a strong signal was observed in the acrosomal cap only in sperm suspensions incubated under conditions to promote capacitation (Figure 3C)

The frequency of each distribution pattern in sperm suspensions recovered from the *cauda* epididymis (non-capacitated), after incubation under conditions to promote sperm capacitation (capacitated), and in those capacitated and then treated with Ca^2+^-ionophore A23187 to induce the AE was evaluated. As summarized in Table 1, acrosome-intact non-capacitated and capacitated spermatozoa showed mainly an E-cad signal in the A + PA distribution pattern, while Ca^2+^-ionophore acrosome-reacted spermatozoa depicted mainly the PA pattern. On the other hand, acrosome intact spermatozoa both non-capacitated and capacitated displayed a N-cad signal in the acrosome in most of the cells, accompanied by a signal in the equatorial segment in around half of the cases (A and A + E patterns), while Ca^2+^-ionophore acrosome-reacted spermatozoa predominantly displayed the ES pattern.

### 3.2. Immunodetection of E-cad and N-cad in Cumulus Oophorus Cells and Oocytes Recovered from Mature COCs

**Figure 4:**
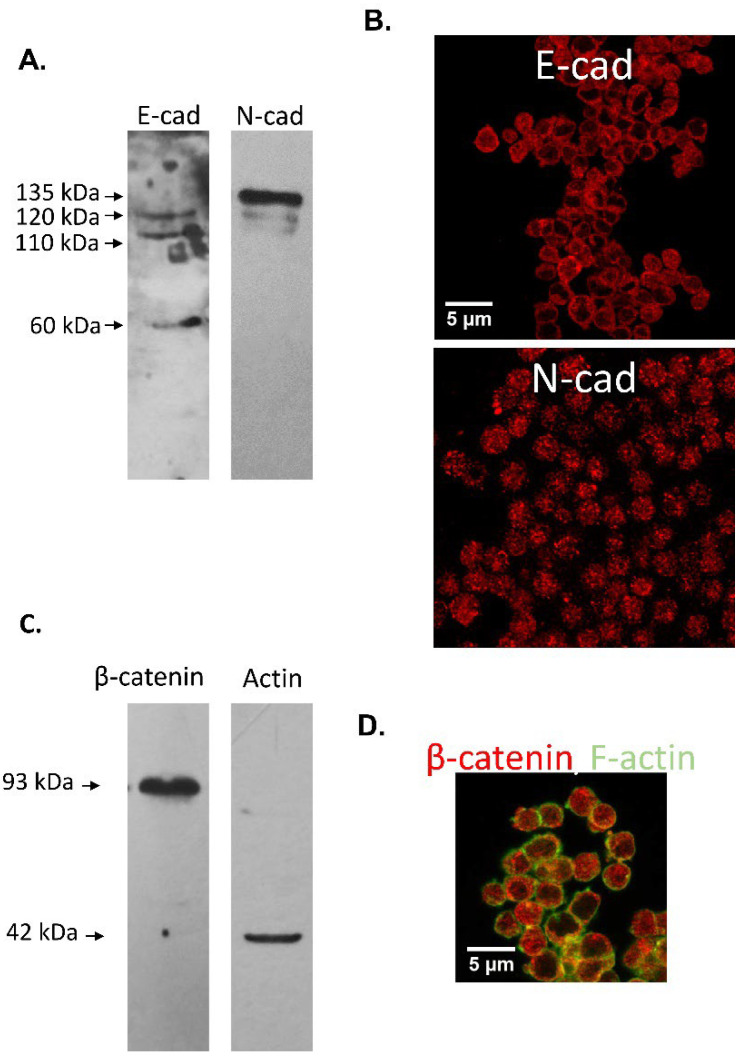
Immunodetection of E-cad, N-cad and related proteins of the adherent complex in mouse cumulus cells. (**A**,**C**). Western immunoblotting of murine cumulus cells with (**A**) anti E-cad and anti N-cad antibodies and (**C**) with antiβ-catenin and anti actin antibodies. Protein forms of 120, 110 and 60 kDa were found for E-cad, while a 135 kDa form for N-cad, a 93 kDa for β-catenin and 42 kDa for actin was found. (**B**,**D**). Fluorescent cytochemistry of fixed cumulus cells using anti E-cad, N-cad and β-catenin antibodies and Phalloidin. E-cad showed a strong signal in the plasma membrane, stronger in cell-cell contacts, accompanied by a homogeneous cytoplasmic signal. N-cad showed a patchy distribution in the cell cytoplasm. β-catenin signal was distributed in the cell cytoplasm and membrane. F-actin signal was mainly localized to the plasma membrane. Bar: 5 µm.

**Figure 5:**
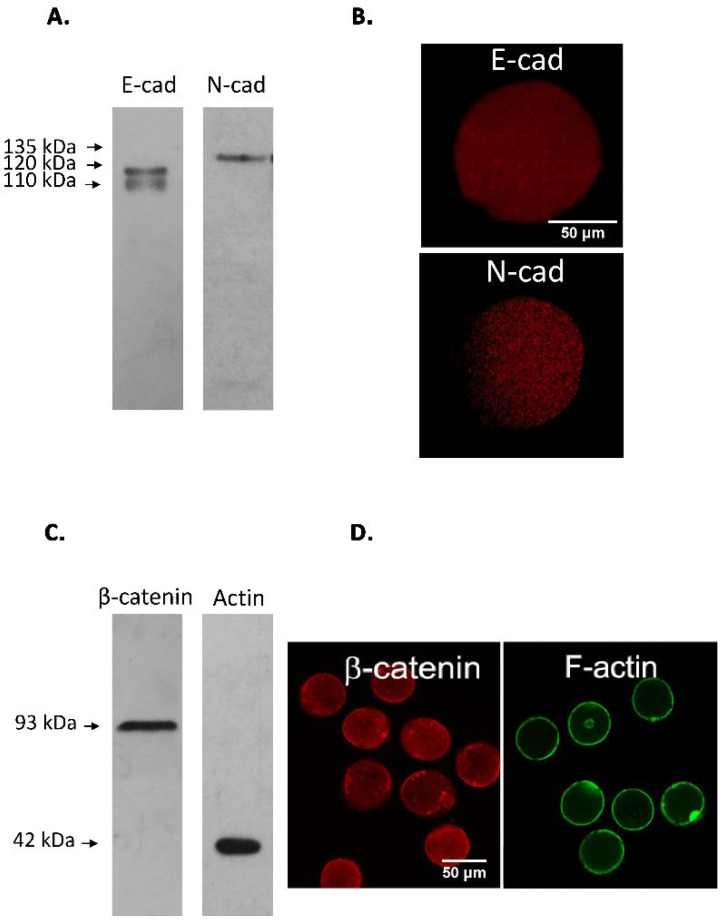
Immunodetection of E-cad, N-cad, and related proteins of the adherent complex in murine oocytes. (**A**,**C**). Western immunoblotting of murine ZP-free oocytes with (**A**) anti E-cad and anti N-cad antibodies, and with (**C**) anti β-catenin and anti actin antibodies. Protein forms of 120 and 110 kDa were found for E-cad, while a 135 and 110 kDa forms for N-cad, a 93 kDa for β-catenin and 42 kDa for actin were found. (**B**,**D**). Fluorescent immunocytochemistry of denuded (devoid of cumulus cells and ZP) oocytes using anti E-cad or anti N-cad antibodies. E-cad signal was homogeneous in the cytoplasm. N-cad signal in the cytoplasm was patchy. β-catenin signal was found in the cell cytoplasm and in some cases along sections of the cell surface. F-actin signal showed the characteristic ring-distribution in the oolemma. Bar: 50 µm.

Protein forms of the expected MW of the adherent complex components were identified in protein extracts of murine cumulus oophorus cells (Figure 4A,C) and oocytes (Figure 5A,C): E-cad (120 kDa), N-cad (135 kDa), β-catenin (92 kDa) and actin (42 kDa). In addition, two E-cad processing products of 110 and 60 kDa were detected in cumulus cells protein extracts and 100 kDa E-cad and N-cad fragments were immunodetected in oocyte protein extracts.

Immunocytochemical analysis of E-cad in cumulus cells showed a homogeneous cytoplasmic signal accompanied by a strong signal in the plasma membrane, stronger in cell-cell contacts. N-cad showed a patchy distribution in the cumulus cells cytoplasm (Figure 4B). While β-catenin signal appeared distributed in the cell cytoplasm and in some cases along the surface, F-actin distribution was mainly localized to the plasma membrane (Figure 4C).

Immunolocalization studies done in cumulus-free mature oocytes without ZP revealed a homogeneous signal for E-cad and a patchy signal for N-cad in the cell cytoplasm (Figure 5B). As observed in cumulus cells, while β-catenin signal was found in the cell cytoplasm and in some cases along sections of the cell surface, F-actin signal showed the characteristic ring-distribution in the plasma membrane (Figure 5C).

### 3.3. Effect of Anti-Cadherins Antibodies and Blocking Peptides upon Murine Fertilization

Immunolocalization studies done in spermatozoa and COCs revealed expression of both E-cad and N-cad in structures involved in sperm-oocyte recognition, leading us to propose the participation of the adhesion proteins in fertilization. To test this, protocols of sperm pre-incubation with specific antibodies towards E-cad (ECCD-1) and N-cad (H-63) followed by gamete interaction assays were implemented. ECCD1 has been previously reported to disrupt cell-cell adhesion when added to monolayer cultures of teratocarcinoma cells [44], and to disarray the compacted morphology of 8- to 16-cell-stage mouse embryos, by blocking E-cad meditated Ca^2+^-dependent cell-cell adhesion between blastomeres [45]. On the other hand, we have previously demonstrated the ability of H-63 anti N-cad antibody to impair human sperm penetration of ZP-free hamster oocytes [30]. In addition to the use of antibodies, specific blocking peptides towards E-cad (EAKYILYSHAVSSNGEAV) were reported to impair MPT cells aggregation [32] (VLYSYHASNIVEKSAGA; scramble control peptide). On the other hand, N-cad blocking peptides (H-SWTLYTPSGQSK-NH2) were tested due to its specific disrupting capacity on N-cad-dependent HUVEC cells aggregation [33] (H-SRTLYTPSGQSK-NH2; scramble control peptide). Furthermore, a dual E-cad/N-cad blocking peptide was tested (H-SWELYYPLRANL-NH2; scramble control peptide: H-SRELYYPLRANL-NH2) given its previously described MDA-MB435 cells aggregation blockage [34]. The use of the blocking peptides rules out any spatial-dependent antibody blockage in the gamete interaction assay.

#### 3.3.1. In Vitro Fertilization 

In vitro fertilization (IVF) procedures were carried out using ovulated COCs or cumulus-free mature oocytes. In assays done using mature COCs, pre-incubation with ECCD-1 anti E-cad or H-63 anti N-cad antibodies resulted in a decreased percentage of fertilized oocytes compared to controls pre-incubated with rat or rabbit IgG added at the same concentration as the specific antibody (Table 2). In line with these findings, in IVF procedures done with oocytes devoid of the cumulus cells, pre-incubation with either ECCD-1 or H-63 antibodies resulted in lower fertilization rates than controls (Table 2).

IVF assays with denuded ZP-free oocytes were also performed, finding significantly lower fertilization rates using either antibodies or blocking peptides (Table 3). E-, N-cad and dual peptides were able to inhibit fertilization of ZP-free oocytes (Table 3).

#### 3.3.2. Cumulus penetration and Sperm-Oocyte Interaction Events

To assess the contribution of cumulus cells to the cadherin-dependent adhesion process, the number of spermatozoa penetrating the cumulus vestment was evaluated. As a result, ECCD-1 but not H-63 significantly reduced the number of spermatozoa penetrating the cumulus (Table 4). This was further confirmed using blocking peptides, where E-cad and dual peptides significantly reduced the number of spermatozoa penetrating the cumulus oophorus. Contrarily, the N-cad blocking peptide did not affect this interaction.

The effect of anti E-cad and N-cad antibodies and blocking peptides upon sperm-oolemma interaction was also investigated by estimating the number of spermatozoa bound to the oolemma. ECCD-1 but not H-63 significantly blocked sperm binding. Nevertheless, all E-cad, N-cad, and E-cad/N-cad dual blocking peptides significantly inhibited sperm-oolemma binding (Table 5). Furthermore, sperm-oocyte fusion was significantly inhibited after sperm pre-incubation with either antibodies or blocking peptides against E- and N-cad (Table 6).

The ability of the anti E-cad or N-cad antibodies or blocking peptides toward each or both adhesion proteins to inhibit sperm-oocyte interaction could not be attributed to a deleterious effect upon sperm motility (subjective evaluation), sperm or oocyte viability, or induction of AE, which were carefully monitored in all assays. No differences were found in these parameters between treated and control conditions (data not shown).

## 4. Discussion

Mammalian fertilization is an intricate process that entails a synchronized series of events that lead to the spermatozoon and oocyte fusion. This process involves sperm and oocyte subcellular structures that participate in cell-cell adhesion events. In this regard, the cadherin superfamily comprises a wide set of adhesion molecules characterized for their relevance in mainly homophilic (same cadherin) Ca^2+^-dependent cell-cell interactions [46,47]. Studies from our group previously described the expression of both E-cad and N-cad in human spermatozoa and oocytes, and reported evidence of their role in sperm-oocyte interaction events [29,30,31]. However, ethical limitations hampered the assessment of human E-cad and N-cad involvement in gamete interaction in a homologous system. The extensive use of the murine model to study mammalian fertilization prompted us to perform a set of studies to assess the involvement of both classical cadherin proteins in adhesion events during gamete interaction leading to fertilization.

Mouse spermatozoa were immunoreactive to E-cad and N-cad in the acrosomal region of acrosome-intact spermatozoa, while Ca^2+^ ionophore acrosome-reacted spermatozoa depicted a signal for E-cad in the post-acrosomal region, and N-cad was mainly distributed in the equatorial segment. A preliminary description of the localization of both adhesion proteins in mouse spermatozoa was previously reported by us [31]. A signal for E-cad in the acrosomal cap had also been previously reported in rats [48] and humans [29]. Moreover, N-cad localization in the equatorial segment of acrosome reacted spermatozoa was previously reported by our group [31]. Participation of the sperm post-acrosomal region in binding/fusion to the oolemma has been proposed in studies done in several species [49,50,51]. In particular, previous reports have described post-acrosomal localization of several sperm proteins highly relevant to fertilization, among them FA-1, CD9 and CD91 osteopontin, ERp29, PLCζ, and PR-R (ProRenin receptor) [52,53,54,55,56,57]. Specifically, FA-1 was proposed to mediate primary sperm-ZP binding in humans and mouse models [52], while CD9 and CD81 are members of the tetraspanin superfamily and were found to participate in sperm–egg membrane fusion [53]. On the other hand, osteopontin was defined as a fertility marker in bull spermatozoa [54], and ERp29 has been associated to gamete interaction, by triggering sperm binding to egg receptors [55]. Finally, PLCz, has been involved in egg activation [56], and higher percentages of PRR-positive spermatozoa were associated with poor sperm motility, and worse blastocyst development [57]. Specifically regarding mechanisms of cell signalling in spermatozoa, Epac1 and 2 proteins were found in spermatozoa, localized in the post-acrosomal region, related to sperm motility, capacitation and AE and associated to E-cad redistribution when activated [58]. With regard to the equatorial segment, it has been widely proposed to contain proteins complexes involved in sperm-oocyte binding and fusion, since several proteins are relocated or exposed at the equatorial segment during sperm capacitation and the acrosome reaction, among them, IZUMO1 [59]. In any case, the entities that participate in this key event of fertilization are still under investigation. In recent years, several proteins were identified using knock-out models, some of which have been proposed to participate in IZUMO1 relocalization and/or cooperate with IZUMO1; among them are SPACA1, TMEM95, FIMP, SOF1 and SPACA6 [60,61,62,63]. Whether E-cad and N-cad are part of a large protein complex that cooperate with IZUMO1 to achieve sperm-oolemma fusion remains to be determined, and may require conditioned tissue specific knock-out models, since homozygous E-cad and N-cad null embryos were previously reported to die early during development [64,65].

Our study showed sperm head actin polymerization under capacitating conditions, and F-actin depolymerization after acrosomal exocytosis, as previously reported [66,67]. F-actin dynamics related to several relevant events of fertilization, among them relocation of IZUMO1 [68], and incorporation of the sperm head into the ooplasm [69]. On the other hand, β-catenin and actin localization follow both adhesion proteins in the acrosomal region after capacitation, results that may suggest a role of the adhesion proteins in other relevant processes leading to fertilization, i.e., IZUMO1 relocation, in addition to support a dynamic adhesive function during gamete interaction reported in other cells [70].

Considering that E-cad and N-cad have been shown to participate in homophilic interactions, its immunolocalization in cumulus cells and the oolemma was investigated, finding protein forms of the expected molecular weight, and members of the adherent complex (β-catenin and actin) in whole protein extracts and whole cells, in agreement with previous studies that describe their expression in human COCs [29,30,48,71,72] and murine cumulus during the germinal vesicle stage [73].

Participation of both cadherins in fertilization was tested using several in vitro gamete interaction assays. Sperm pre-incubation with ECCD-1, a specific monoclonal antibody towards mouse E-cad resulted in a significant inhibition of IVF, cumulus penetration and oolemma binding/fusion. This blockage may be due to the ability of ECCD-1 to recognize an epitope from the extracellular E-cad domain, preventing its homotypic dimerization [74]. Since this antibody binds only to the active protein [44], these results are indicative of a functional E-cad adhesion molecule in both cell membranes. Regarding N-cad blocking assays, sperm incubation with the H-63 specific antibody resulted in decreased COCs and oocytes fertilization rates, as well as sperm-oocyte fusion. On the other hand, sperm-oolemma binding was not affected when sperm cells were incubated with anti N-cad antibody. To rule out a possible steric blockage by the antibody, IVF and gametes interaction assays were also performed in the presence of E-cad and N-cad blocking peptides. As a result, cumulus penetration, oolemma binding, sperm-oocyte fusion and fertilization rates were found significantly decreased, confirming the involvement of both E-cad and N-cad-dependent adhesion events leading to fertilization.

Although cell-cell fusion is a rare event for most cell types, it is not restricted to sperm-oocyte interaction. Among other examples, skeletal muscle regeneration involves the arrangement of a fused network of cells that requires differentiation and maturation of muscle progenitor cells. This process requires cell-cell adhesion and communication, and N-cad is one of the cadherins composing the adhesion junctions between myoblasts that regulate cell fusion [75]. N-cad and M-cad (muscle cadherin) have been reported to tether myoblasts to produce tension and mechanotransductive signals to induce fusion [76]. Moreover, cadherins also facilitate gap junction formation when associated to connexins in other cell types [75,77]. Similarly, placental development involves the generation of a multinucleated structure called the syncytiotrophoblast, composed of multiple nuclei that share one common cytoplasm. This structure results from the trophoblast syncytialization, and it involves the regulation of intercellular adherens junctions, where cadherins take part [78].

Cell fusion requires a set of proteins, each one participating in a particular step of the process. These molecules have been grouped in three categories: (1) molecules that elicit initial adhesion, (2) molecules that rearrange the plasma membrane and promote the formation of pores, and (3) molecules that complete the plasma membrane disassembly and conclude cell fusion. In this regard, cadherins participation in adhesion events prior and essential for cell fusion has been reported in somatic and embryonic cells; as an example, in trophoblast syncytialization [79]. This cadherin-mediated cell-cell adhesion event is regulated by E-cad proteolysis of the extracellular domain triggered in association with an increase in Ca^2+^ ions efflux [79,80,81], activation of several signaling pathways that trigger its re-localization to intracellular compartments followed by degradation or recycling, N-cad intracellular transport via KIF3A-bound cargo vesicles [82], and internalization involving phosphorylation of Tyr residues by several kinases leading to endocytosis of the complex [18]. Alternatively, E-cad adhesive function may also be altered through gene regulation. In this regard, β-catenin involvement in gamete adhesion and fusion was reported using genetic modified oocytes [83]. In somatic cells, β-catenin translocation to the cell nucleus triggers profound changes in gene expression, particularly the epithelial-to-mesenchymal transition (EMT), a crucial process for embryogenesis and organ morphogenesis, wound healing, and malignant progression [84,85,86]. The EMT process involves remodelling of cell-cell and cell-extracellular matrix interactions, leading to the loss of cell polarity and cell-cell adhesion properties among epithelial cells. The molecular events involved in EMT are highly complex, and comprise a decrease in expression of epithelial markers, such as E-cad, accompanied by an increase in the expression of mesenchymal markers, such as N-cad, changes that provide cell plasticity and result in increased cell migration and invasion properties. EMT-related events have been fully characterized in early embryogenesis [87,88]. An EMT-like process may, at least in part, be responsible for the progression from adhesion to fusion in gamete interaction, considering the striking similarities to these mechanisms already identified in gametes.

## 5. Conclusions

**Figure 6:**
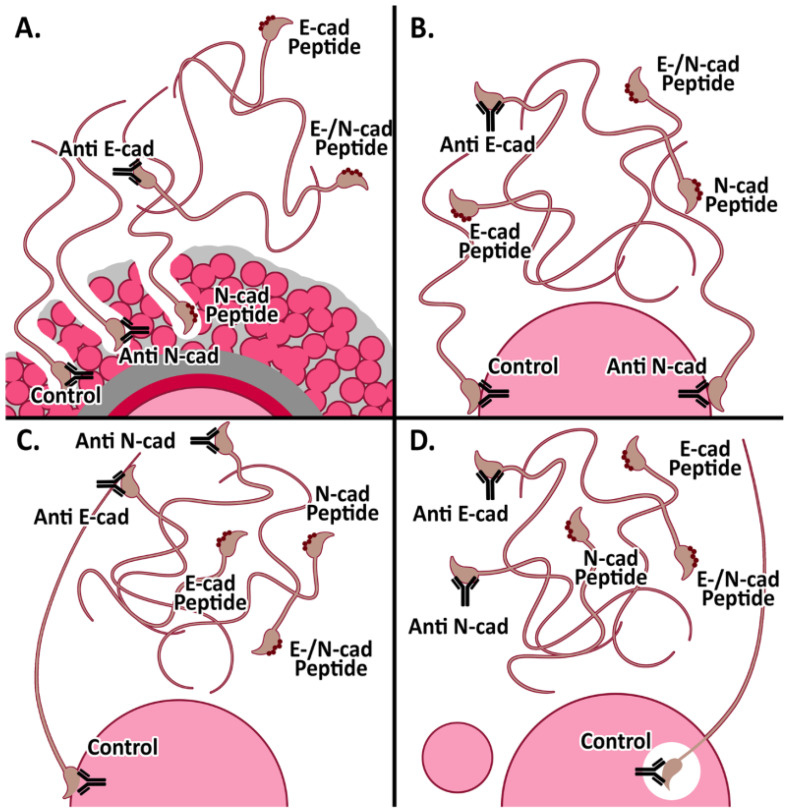
Schematic representation of E-cad and N-cad participation in murine fertilization. (**A**). Cumulus penetration assay: sperm-cumulus interaction was significantly impaired after sperm pre-incubation with anti E-cad antibodies as well as peptides towards E-cad alone or E-/N-cad dual peptide. (**B**). Oolemma binding assay: sperm-oolemma interaction was inhibited after sperm pre-incubation with anti E-cad antibody and peptides towards each E-cad and N-cad, or dual to both E-cad/N-cad. (**C**). Fusion assay and (**D**). IVF assay: sperm-oocyte fusion and fertilization were inhibited after sperm pre-incubation with either E- or N-cad antibodies and blocking peptides towards E-, N- or both E-cad/N-cad.

The studies presented in this report have thoroughly evaluated the localization of E-cad, N-cad, and other members of the adherent complex in mouse gametes, and showed evidence on the involvement of both classical cadherins in gamete interaction events leading to fertilization. Figure 6 shows a schematic representation of the findings presented in this report. Our results in the murine model confirm the observations previously described in the human model, and provide biochemical and molecular tools to further characterize the mechanisms underlying participation of E-cad and N-cad in mammalian fertilization. Understanding this fascinating process will contribute to basic gamete physiology and ultimately may help to the diagnosis and treatment of male infertility.

## Figures and Tables

**Table 1:** Immunolocalization patterns of E-cad and N-cad adhesion proteins in murine spermatozoa.

**A.**			
**E-cad**	**A + PA**	**PA**	**N**
Intact Non-Capacitated	90.3 ± 5.7	8.0 ± 4.6	1.7 ± 1.2
Intact Capacitated	69.0 ± 6.0	22.7 ± 6.7	8.3 ± 1.5
A23187 Ca^2+^-ionophore acrosome-reacted	20.0 ± 2.7	75.3 ± 7.4	4.7 ± 4.7
**B.**			
**N-cad**	**A**	**A + ES**	**ES**
Intact Non-Capacitated	39.8 ± 19.8	51.8 ± 15.6	8.4 ±6.0
Intact Capacitated	40.1 ± 17.9	40.6 ± 12.1	19.3 ± 6.0
A23187 Ca^2+^-ionophore acrosome-reacted	15.9 ± 5.2	17.2 ± 7.7	66.9 ± 10.0

A. In adult CF1 male mice, E-cad was immunolocalized in the acrosomal and post-acrosomal regions (A + PA), post-acrosomal region (PA) or showed no signal (N). Non-capacitated and capacitated intact spermatozoa mainly showed the A + PA localization pattern, while Ca^+2^-ionophore A23187acrosome-reacted spermatozoa predominantly depicted the PA pattern. B. N-cad was immunolocalized in the acrosomal region (A), in the equatorial segment (ES), or in both subcellular regions (A + ES). Non-capacitated and capacitated intact spermatozoa showed mainly the A + ES immunolocalization pattern and the A pattern, while Ca^+2^-ionophore A23187 acrosome-reacted spermatozoa predominantly had the ES pattern. Results are expressed as Mean ± Standard Deviation of the Mean (SDM).

**Table 2:** IVF assays with COCs or cumulus-free oocytes using anti E-cad or N-cad antibodies.

IVF	Control (%)	Pre-Treated (%)
COC E-cad Antibody	66.6 ± 4.4 (*n* = 117)	31.8 ± 7.2 * (*n* = 123)
N-cadAntibody	43.8 ± 9.1 (*n* = 29)	14.3 ± 8.3 ** (*n* = 40)
Cumulus-free Oocytes		
E-cad Antibody	57.5 ± 36.5 (*n* = 93)	11.0 ± 1.6 * (*n* = 95)
N-cad Antibody	56.4 ± 6.1 (*n* = 30)	13.9 ± 2.8 ** (*n* = 30)

Percentage (%) of fertilized oocytes after sperm pre-incubation with anti E-cad (ECCD-1) or anti N-cad (H-63) antibodies. IVF done with COCs or oocytes devoid of cumulus cells (Cumulus-free Oocytes) was significantly impaired after incubation with either anti E-cad or N-cad antibodies. Results are expressed as mean percentage of fertilized oocytes ± SEM. * *p* < 0.05 ** *p* < 0.005 (Chi-squared test). The number of COCs or cumulus-free oocytes used in each condition is placed in parenthesis.

**Table 3:** IVF assays of denuded oocytes with anti E-cad or N-cad antibodies and blocking peptides towards each of both adhesion proteins.

ZP-Free Oocytes IVF	Control (%)	Pre-Treated (%)
E-cad Antibody	58.1 ± 1.0 (*n* = 24)	24.5 ± 2.5 * (*n* = 25)
N-cadAntibody	72.6 ± 6.3 (*n* = 29)	27.8 ± 5.6 ** (*n* = 30)
E-cad Peptide	50.9 ± 4.8 (*n* = 49)	18.1 ± 5.9 *** (*n* = 55)
N-cadPeptide	57.5 ± 8.5 (*n* = 48)	24.3 ± 8.2 ** (*n* = 46)
E-/N-cad Dual Peptide	69.1 ± 6.4 (*n* = 51)	22.8 ± 8.6 *** (*n* = 45)

Percentage (%) of fertilized oocytes after sperm pre-incubation with anti E-cad (ECCD-1) or anti N-cad (H-63) antibodies or blocking peptides. IVF of ZP-free oocytes was significantly impaired after sperm pre-incubation with specific antibodies towards E-cad (ECCD-1) or N-cad (H-63), as well as blocking peptides towards each adhesion protein or both E-cad or N-cad adhesion proteins. Results are presented as the mean percentage of fertilized denuded oocytes ± SEM. * *p* < 0.05 ** *p* < 0.005 *** *p* < 0.0005 (Chi-squared test). The number of denuded oocytes used in each condition is placed in parenthesis.

**Table 4:** Cumulus Penetration Assay using anti E-cad or N-cad antibodies or blocking peptides towards each of both adhesion proteins.

Cumulus Penetration Assay	Control (# Spermatozoa)	Pre-Treated (# Spermatozoa)
E-cad Antibody	32.9 ± 2.0 (*n* = 16)	16.8 ± 1.0 *** (*n* = 13)
N-cad Antibody	32.9 ± 2.0 (*n* = 16)	29.8 ± 2.3 (*n* = 17)
E-cad Peptide	38.3 ± 2.0 (*n* = 32)	27.3 ± 2.9 ** (*n* = 29)
N-cad Peptide	32.1 ± 7.0 (*n* = 27)	31.9 ± 14.5 (*n* = 30)
E-cad/N-cad Dual Peptide	43.3 ± 2.4 (*n* = 28)	33.6 ± 2.2 * (*n* = 25)

The number (#) of spermatozoa penetrating the cumulus mass after sperm pre-incubation with anti E-cad (ECCD-1) or anti N-cad (H-63) antibodies or blocking peptides toward each of both adhesion proteins. Cumulus penetration was significantly impaired after sperm pre-incubation with anti-E-cad antibody (ECCD-1) or blocking peptides towards E-cad, N-cad or both E-/N-cad. Results are presented as the mean number of spermatozoa penetrating the cumulus cells layer ± SEM. * *p* < 0.05 ** *p* < 0.005 *** *p* < 0.0005 (Mann–Whitney test). The number of cumulus masses used in each condition is placed in parenthesis.

**Table 5:** Oolemma binding assay using anti E-cad or N-cad antibodies or blocking peptides towards each of both adhesion proteins.

Oolemma Binding	Control (# Spermatozoa)	Pre-treated (# Spermatozoa)
E-cad Antibody	25.8 ± 1.1 (*n* = 70)	11.5 ± 1.0 *** (*n* = 60)
N-cad Antibody	18.9 ± 1.6 (*n* = 54)	15.8 ± 1.3 (*n* = 63)
E-cad Peptide	9.0 ± 0.8 (*n* = 50)	2.4 ± 0.6*** (*n* = 52)
N-cad Peptide	14.2 ± 1.3 (*n* = 59)	6.8 ± 1.7*** (*n* = 54)
E-cad/N-cad Dual Peptide	14.5 ± 1.2 (*n* = 56)	3.6 ± 0.6*** (*n* = 56)

The number (#) of sperm binding to the oolemma after sperm pre-incubation with anti E-cad (ECCD-1) or anti N-cad (H-63) antibodies, as well as blocking peptides toward each of both adhesion proteins. Oolemma binding was significantly impaired after sperm-pre-incubation with specific antibodies towards E-cad or peptides towards or E-cad, N-cad or both E-cad/N-cad. Results are presented as the mean number (#) of spermatozoa bound to the oolemma of ZP-free occytes ± SEM. *** *p* < 0.0005 (Mann–Whitney test). The number of ZP-free oocytes used in each condition is placed in parenthesis.

**Table 6:** Sperm-oocyte fusion assay using anti E-cad or N-cad antibodies or blocking peptides toward each of both adhesion proteins.

Sperm-oocyte Fusion	Control (%)	Pre-Treated (%)
E-cad Antibody	91.3 ± 4.2 (*n* = 52)	24.3 ± 14.6 *** (*n* = 40)
N-cad Antibody	73.7 ± 3.5 (*n* = 44)	44.3 ± 9.9 * (*n* = 63)
E-cad Peptide	76.5 ± 7.6 (*n* = 50)	33.9 ± 7.5 *** (*n* = 52)
N-cad Peptide	81.0 ± 6.6 (*n* = 59)	28.3 ± 4.8 *** (*n* = 54)
E-cad/N-cad Dual Peptide	75.9 ± 2.2 (*n* = 56)	25.2 ± 10.3 *** (*n* = 56)

Percentage (%) of fertilized oocytes after sperm pre-incubation with anti E-cad (ECCD-1) or anti N-cad (H-63) antibodies, as well as blocking peptides toward each of both adhesion proteins. Sperm-oocyte fusion was significantly impaired after sperm pre-incubation with specific antibodies or blocking peptides. Results are presented as the mean percentage of fused oocytes ± SEM. * *p* < 0.05 *** *p* < 0.0005 (Chi-squared test). The number of ZP-free oocytes used in each condition is placed in parenthesis.

## Data Availability

The raw data presented in this study are available on request from the corresponding author.
